# Development and validation of the multi-dimensional health resilience scale for community-dwelling adults

**DOI:** 10.3389/fpubh.2025.1452738

**Published:** 2025-02-12

**Authors:** Lixia Ge, Wan Fen Yip, Ruijie Li, Eric Siang Seng Chua, Moon-Ho R. Ho, Andy Hau Yan Ho, Evon Yiwen Chua, Dolly Cheng, Ian Yi Onn Leong, Pann Pei Chieh, Woan Shin Tan

**Affiliations:** ^1^Health Services and Outcomes Research, National Healthcare Group, Singapore, Singapore; ^2^School of Social Sciences, Nanyang Technological University, Singapore, Singapore; ^3^Lee Kong Chian School of Medicine, Nanyang Technological University, Singapore, Singapore; ^4^Population Health & Community Transformation, Yishun Health, Singapore, Singapore; ^5^Clinical Operations (Integration Care), Woodlands Health, Singapore, Singapore; ^6^Continuing and Community Care, Tan Tock Seng Hospital, Singapore, Singapore

**Keywords:** health resilience, instrument development, scale, measure, assessment, psychometric properties, validation

## Abstract

**Introduction:**

Resilience measures generally are not health specific, nor do they account for the multiple dimensions required for individuals to overcome health challenges. To bridge this gap, we developed and validated a multi-dimensional Health Resilience Scale (HRS) for community-dwelling adults in Singapore.

**Methods:**

We followed standard procedures to develop health resilience construct, identify dimensions, and generate potential items. Expert review and cognitive interviews were conducted to assess content validity and item clarity. The refined 35-item HRS was administered to 650 eligible community-dwelling adults in a cross-sectional survey, along with validation measures, to assess construct validity (including factorial, concurrent, convergent, and divergent validity) and internal consistency reliability.

**Results:**

Exploratory factor analysis revealed five factors with 22 items, each factor containing 3 to 5 items. Confirmatory factor analysis confirmed the five-factor structure with good model fit. The five factors identified in the analysis were conceptualised as the following dimensions of the HRS: “Health mindset,” “Perceived health access,” “Social resourcefulness,” “Relational support,” and “Adaptive adjustment.” The dimensions of “Health mindset,” “Perceived health access,” and “Adaptive adjustment” exhibited moderate and positive correlations with psychological resilience (concurrent validity) as well as hope and self-efficacy (convergent validity). All dimensions had weak or no correlation with maladaptive coping, depression, and anxiety measures (divergent validity). Individuals with better health status scored higher, while those with recent health adversity scored lower on the HRS, confirming divergent validity. Internal consistency reliability was confirmed with Cronbach’s alpha exceeding 0.80 for the total scale and ranging from 0.73 to 0.88 for individual dimensions.

**Conclusion:**

The 22-item multi-dimensional HRS demonstrated good reliability and validity, making it an effective tool for assessing health resilience and guiding initiatives aimed to enhance well-being among community members.

## Introduction

Strengthening resilience is a key priority within the European policy framework of the World Health Organisation, where the emphasis is on fostering positive health and well-being (1). Similarly, strengthening resilience is also the backbone of Singapore’s healthcare system transformation, where concerted efforts to establish a sustainable care ecosystem capable of addressing the complexities and challenges stemming from a rapidly aging population are underway. The pivotal elements of its health management strategy includes the development of community-based platforms geared towards enhancing health literacy, nurturing healthy behaviours, and fostering neighbourhood social cohesion (2). Such endeavours have catalysed the adoption of innovative strength-based approaches aimed at bolstering resilience among both individuals and communities.

Following highly stressful events, individuals demonstrate varying capacities to adapt, with some smoothly returning to healthy functioning while others grapple with significant distress (3). This dichotomy underscores the necessity for a thorough examination of approaches to identify and support individuals pre-event, so that their coping abilities against future stressors can be strengthened. Such an endeavour requires a thorough understanding of an individual’s resilience in the face of health-related adversities as well as the delineation of its fundamental processes from a holistic perspective, grounded in cultural and contextual sensitivity.

Recent empirical interest has been drawn to unravelling the significance of protective factors and the mechanisms that underpin individuals’ recovery from adversity, thus maintaining a high level of well-being and quality of life (4). Research indicates that psychological attributes such as self-adjustment, optimism, and positive emotions, along with social factors like perceived support and connectedness, as well as physical characteristics such as independence in activities of daily living, significantly predict resilience in the adult population (4). Furthermore, the ability to “bounce back” from adversity is also influenced by the availability of various contextual resources that promote resilience (5).

Taking a life course perspective and embedding the concept within a socio-ecological framework, Benett and Windle conceived individual resilience as a dynamic interplay of resources across the individual, community, and societal levels during challenging health adversity (6). This comprehensive framework acknowledges the collective influence of individual-level factors (e.g., personal characteristics such as beliefs, optimism, and sense of self-efficacy), community resources, and societal forces in shaping resilience. Embracing the socio-ecological perspective reveals that nurturing an individual’s resilience necessitates not only considering their personal traits but also ensuring the availability and accessibility of relevant resources and support systems within their environment (7, 8).

Windle’s concept analysis (8) of resilience suggest that providing suitable resources (e.g., enhanced services and treatment and altered contextual factors) may help mitigate or prevent negative outcomes resulting from stressors or adversities. Additionally, her theoretical exploration highlights that resilience is intertwined with everyday life. This underscores the relevance of resilience building for all individuals, not solely those who have experienced risk or adversity.

Many tools have been developed to assist practitioners and policymakers in monitoring the progress of programmes aimed at promoting and strengthening resilience while identifying potential gaps in resilience-promoting efforts. In recent years, several multi-dimensional resilience tools have emerged, focusing on personal characteristics, attitudes, trust, commitment, coping abilities, adaptability, social support, and/or quality of social relationships. Despite these advancements, none have comprehensively captured the significant role of social resources and support systems on the process or as a mechanism of resilience. Recognising that resilience in the face of health adversities extends beyond individuals’ personality traits and acknowledging the supportive role of interventions and health-friendly environments in empowering individuals and communities to rebound from health-related challenges (9), it is imperative to assess how an individual’s connection to or ability to utilise system-level factors (e.g., public assistance programmes, healthcare financing policies) may impact their access to resources (e.g., health or social care services) and, consequently, their health resilience.

It is noteworthy that most existing resilience tools were developed for North American or European populations within Western societal contexts, thus lacking representation from an Asian perspective. Resilience research underscores both commonalities and differences across cultural groups concerning coping strategies and access to health resource (10). Culture and contextual nuances can give rise to variations in how individuals negotiate and interact with their community and environments. Consequently, there is a compelling need to address the dearth of culturally relevant research in health adversity-related resilience.

Hence, we conducted this multi-stage study to develop a multi-dimensional health resilience scale (HRS) for assessing an individual’s capability to rebound from health-related challenges or stressors and to examine its psychometric properties, including construct validity and internal consistency reliability, in a sample of English-speaking community-dwelling adults in Singapore. The research questions are:

1. What are the key dimensions of health resilience in community-dwelling adults across diverse demographic and health backgrounds?
2. What items should be included in the self-report HRS to adequately measure health resilience in community-dwelling adults?
3. What is the factor structure of the scale, and how many factors (dimensions) are revealed through exploratory and confirmatory factor analysis?
4. What is the internal consistency of the scale (e.g., Cronbach’s alpha) for assessing health resilience in community-dwelling adults?
5. Does the HRS demonstrate good construct validity, including concurrent, convergent, and divergent validity?

## Methods

### Study setting and participant recruitment for validation

Community-dwelling adults were conveniently recruited at various locations, including Community Health Posts and health-related public events. Additional recruitment methods included promotional posters, participant referrals, and word-of-mouth. Eligibility criteria were: (1) age 21 years or older; (2) Singapore citizen or permanent resident; and (3) ability to independently read, comprehend, and complete an English-language survey. Since health resilience is a relevant construct for people with diverse health backgrounds, the HRS was intentionally developed for use across the health spectrum, including both healthy individuals and those with various health conditions. As such, the recruitment of the validation sample was not limited to healthy adults.

Candidates were either contacted via their registered phone numbers or approached in-person by trained surveyors at recruitment sites. After introducing the study and conducting an eligibility screening, eligible candidates were scheduled for a face-to-face survey. Written consent was obtained in person after ensuring that the participants fully understood the study’s purpose, procedures, and their responsibility. Subsequently, participants were asked to independently complete the survey, which included demographics, potential HRS items and validation measures. Compensation in the form of shopping vouchers was provided to each participant upon survey completion.

The sample size was determined based on recommendations for exploratory factor analysis (EFA) and confirmatory factor analysis (CFA). Given the minimum suggested sample size of 10 participants per item for EFA (11, 12) and 300 for a population model for CFA (13), the targeted sample size was set at 650, with 350 for EFA and 300 for CFA.

### Development of the health resilience scale

In this study, we adopted a modified version of Gill Windle’s definition of resilience concerning stressful health-related adversities (8), characterising health resilience as “the dynamic process of positively negotiating, regulating, adapting to, and managing significant sources to strive despite health-related stress or adversity.” This definition underscores the role of behavioral and psychological regulation, assets and resources within individuals, their lives, and environments in facilitating effective adaptation and ‘bouncing back’ in the face of health-related adversity.

The development of the HRS was carried out in four distinct stages:

Stage 1: Identifying the domains of health resilience

This stage involved a comprehensive process of identifying the core domains and processes of health resilience. This included conducting a thorough literature review, conducting in-depth semi-structured interviews with 50 community-dwelling individuals facing health adversities, interviewing 14 caregivers providing care to family members with health adversities, and facilitating 11 focus group discussions with a total of 53 community health and social care providers. Through the analysis of transcripts from these various sources, seven core domains emerged for health resilience, including (1) interpersonal protective factors, (2) self-adjustment ability, (3) health literacy, (4) healthcare affordability, (5) perception of medical services, (6) perception of social services, and (7) supportiveness of social networks. These seven domains informed the development of the preliminary measure.

Stage 2: Drafting the initial version of the HRS

This stage, encompassing item bank generation, internal review, item refinement, expert review, and subsequent refinements upon discussions, is to operationalise the seven dimensions through survey items. The item bank comprised 313 items, which were either developed based on Stage 1 findings or adapted from existing resilience measures. Following internal review and item refinement, 60 items remained across the seven dimensions.

Stage 3: Refining HRS items through expert review

The 60 items, together with the proposed dimensions, were sent to eight identified local and international experts for review. These experts were professors or senior researchers specialising in health promotion and motivation, psychological resilience research, mental health, ageing, and social health. Their ratings and feedback for both dimensions and items were meticulously summarised in Appendix 1, and items were further refined through iterative discussions. Ultimately, this process yielded 35 items within seven proposed dimensions. Response options for each item were provided on a 5-point Likert scale, ranging from 1 (Strongly Disagree) to 5 (Strongly Agree), with intermediate options for various levels of agreement or disagreement.

Stage 4: Refining HRS items through cognitive interviews

Cognitive interviews were conducted to assess the readability, clarity, and comprehensibility of the 35 items. A survey form, including these items along with instructions, was developed. Twenty community-dwelling adults were purposively recruited to independently complete the survey with virtual interviewers’ presence. Among the participants, six (30%) were aged 21–30 years and another six were aged 31–40 years. Four participants each were in the 41–60 years and 60 years and above age groups. Twelve (60%) were females and 17 (85%) were Chinese. Five participants had lower secondary to post-secondary education background, while the remaining were diploma holders or had higher education. The majority (65%) had experienced health adversity. Subsequently, cognitive interviewing sessions were conducted to evaluate participants’ overall impression of the scale, item readability and interpretation, and appropriateness of response options. Moreover, the time taken to complete the measure and its suitability for respondents without health adversities were tested. Following the identification of issues and input from participants, we refined the wording of 24 items, deleted one item, and introduced one new item. The refined set of 35 items (see Appendix 2) was then integrated into the survey questionnaire, alongside socio-demographics and validation measures, for data collection.

### Measures for validity testing

To comprehensively assess the validity of the HRS, several validated measures were purposefully selected. These measures were chosen to explore various aspects of validity, including concurrent, convergent, and divergent validity, providing a robust evaluation of the HRS’s effectiveness in measuring the intended constructs.

#### The Connor-Davidson resilience scale 10-item

As a widely used unidimensional measure to quantify self-perceived resilience, the Connor-Davidson Resilience Scale 10-item (CD-RISC10) was selected as the “gold standard” for measuring resilience and used for examining the concurrent validity of the HRS, a subtype of criterion validity (14). Adapted from the original 25-item CD-RISC (15, 16), the CD-RISC10 is renowned for its reliability and validity established across various age groups, clinical and general populations, and diverse linguistic contexts (16–20). Each item is rated on a 5-point Likert scale ranging from 0 (not true at all) to 4 (true nearly all the time). The total score, computed by summing the 10 individual item scores, spans from 0 to 40, with a higher score denoting a higher level of resilience. The Cronbach’s alpha of the CD-RISC10 in this study was 0.92.

#### Herth Hope Index

Hope has been conceptualised as “a positive motivational state that is based on an interactively derived sense of successful agency (goal-directed energy) and pathways (planning to meet goals)” (11). Hope and resilience are considered intricately linked constructs, as they both entail a propensity for maintaining an optimistic perspective amid challenges (12, 13).

The Herth Hope Index (HHI), developed by Herth (21), was included to evaluate the concurrent validity of the HRS. This self-report instrument comprises 12 items, each rated on a 4-point Likert scale ranging from 1 (strongly disagree) to 4 (strongly agree). The overall score, calculated by summing individual item scores (with reversed scores for items 3 and 6), ranges from 12 to 48, with higher scores indicating greater levels of hope. The HHI has been validated in both general and clinical populations (22–25). In this study, the HHI demonstrated good internal consistency reliability, with a Cronbach’s alpha of 0.84.

#### General Self-Efficacy Scale

Bandura’s concept of self-efficacy is widely integrated into the resilience literature and provides a theoretical lens through which to understand the construct of resilience (26, 27). Self-efficacy refers to individuals’ beliefs concerning their capability and capacity to perform behaviours necessary for achieving specific outcomes. Higher self-efficacy scores are typically correlated to greater resilience, better problem-solving abilities, and a more optimistic outlook on life.

To assess the convergent validity of the HRS, the General Self-Efficacy Scale (GSE) was chosen. The GSE is a well-established and reliable instrument to access an individual’s general belief in their ability to handle a variety of challenges and difficult demands in life (28). It comprises10 items with a 4-point Likert scale: 1-not at all true, 2-hardly true, 3-moderately true, and 4-exactly true. The total score, ranging from 10 to 40, is derived by summing the scores of individual items, with higher scores indicating greater self-efficacy. The Cronbach’s alpha of the GSE was 0.87, demonstrating good internal consistency reliability in this study population.

#### Brief-Coping Orientation to Problems Experienced Inventory

Extensive research has explored the relationship between different coping strategies and resilience, highlighting that coping and the concept of resilience are linked but distinct constructs (29, 30). It is evident that adaptive coping strategies are positively correlated with resilience whereas maladaptive coping strategies are not (31, 32).

To assess both adaptive and maladaptive coping strategies, the 28-item Brief-Coping Orientation to Problems Experienced Inventory (Brief-COPE) was utilised, capturing coping strategies across 14 facets (33). We hypothesised that adaptive coping strategies would positively correlate with resilience, while maladaptive strategies would exhibit a negative correlation with resilience. Respondents rated the frequency of using each coping strategy when faced with specific stressful situations on a 4-point Likert scale ranging from 1 (I have not been doing this at all) to 4 (I’ve been doing this a lot). Each subscale score was derived by summing responses to relevant items and then dividing by the number of items within that subscale. The Cronbach’s alpha values for the three coping strategies in this study were 0.87, 0.92, and 0.71, respectively.

#### Patient Health Questionnaire-9

Resilience and depression are distinct constructs with unique conceptualisations. The Patient Health Questionnaire-9 (PHQ-9), a widely used self-report questionnaire specifically designed to screen and assess the severity of depression in individuals, was included to examine the divergent validity of the HRS. The nine items of the PHQ-9 assess the presence and severity of common depressive symptoms experienced over the past two weeks, aligning with the nine criteria used for diagnosing major depressive disorder (34). Individuals rate the frequency of each symptom during this timeframe on a scale ranging from 0 (not at all) to 3 (nearly every day). The total score, ranging from 0 to 27, is generated by summing the scores for individual items, with higher scores indicative of more pronounced depressive symptoms. The PHQ-9 demonstrated good internal consistency reliability (Cronbach’s alpha = 0.83) in the study population.

#### Generalised Anxiety Disorder-7

The Generalised Anxiety Disorder 7-item (GAD-7), a widely used screening tool to assess the severity of general anxiety symptoms across various populations (35), was included as an additional measure to evaluate the divergent validity of the HRS. The GAD-7 prompted individuals to rate the frequency and intensity of anxiety symptoms experienced over the past two weeks using a 4-option scale ranging from 0 (not at all) to 3 (nearly every day). Total scores of the GAD-7 ranged from 0 to 21, with higher scores indicating more pronounced anxiety. The GAD-7 exhibited strong internal consistency reliability in this study, with a Cronbach’s alpha of 0.91.

#### Socio-demographics and health state

Participants’ socio-demographic information including age group, gender, ethnicity, marital status, highest education attained, housing type, living arrangement, employment status, perceived money sufficiency for basic living was collected during the survey. In addition, participants’ self-perceived physical and mental health statuses were obtained by asking, “In comparison with other people of the same age, how do you consider your physical/mental health status” with response options: “Not as good,” “Do not know,” “As good,” and “Better.”

#### Psychometric analyses

The psychometric evaluation of the potential HRS encompassed an examination of item responses as well as an assessment of construct validity and reliability. The construct validity of the scale was scrutinised through factorial validity (the structure of the measure) involving both EFA and CFA, concurrent validity, convergent and discriminant validity. Reliability was established through the examination of internal consistency reliability.

#### Item-level descriptive analysis

The distribution of individual response options for each of the 35 items was described using frequency (n) and percentage (%), accompanied by mean, standard deviation (SD), median, quartile 1 (Q1), and quartile 3 (Q3).

#### Exploratory factor analysis

The EFA utilised data from a randomly selected 350 participants. Prior to the extraction of the constructs, Kaiser-Meyer-Olkin (KMO) and Bartlett’s Test were conducted to assess sample adequacy and data suitability for factor analysis (36). A KMO correlation above 0.70 was considered indicative of sample adequacy for factor analysis, while a *p*-value of <0.05 for Bartlett’s test of Sphericity ensured the appropriateness of factor analysis (37). The correlation matrix was inspected for the relationships between items.

To determine the optimal number of factors to retain, Horn’s Parallel Analysis (PA) for principle components (38), deemed the best method to extract factors (39), was employed in conjunction with Velicer’s minimum average partial (MAP) correlation (40) and the common Kaiser’s eigenvalue >1 criterion for accuracy (41). EFA employing principal-component factor (PCF) analysis followed by the Promax method for oblique rotation was utilised to explore the structure of the proposed HRS and identify underlying unobservable latent factors explaining most variance of these directly observable variables or items (41). The alignment of the factors that items loading onto with the proposed dimensions of the items, the rotated factor loadings, the contents of items that loaded onto unexpected factors were reviewed to determine whether the factor structure was theoretically sound. Items that were theoretically irrelevant to the underlying factors, loaded onto multiple factors, had a factor loading below 0.40, or those demonstrating high correlation (rho>0.80) with another item, thus being considered redundant, were systematically eliminated. Any adjustment to the items included for the analysis prompted a re-conduction of PA, MAP as well as EFA. The rotated factor loading coefficients exceeding 0.30 for individual items in the final EFA were reported.

#### Confirmatory factor analysis

CFA, executed on data from the remaining 300 participants, aimed to validate the factor structure identified in the EFA. Items loaded onto the same factor in the final EFA results were allowed to load onto the corresponding latent factor in the CFA model. Maximum likelihood method was used for estimation. Each latent factor’s variance was set to 1 when constructing the model and standardised factor loadings were generated for individual items within each latent factor.

Model fit was assessed using several fit statistics against predefined criteria: a chi-square to degrees of freedom ratio (χ^2^/df) of 3 or lower, a Root Mean Square Error of Approximation (RMSEA) of 0.06 or lower (42), a Comparative Fit Index (CFI) and Tucker-Lewis Fit Index (TLI) of 0.90 or higher, and a Standardised Root Mean Square Residual (SRMR) of 0.08 or lower (43).

#### Concurrent, convergent, and discriminant validity

To evaluate the concurrent, convergent, and divergent validity of the HRS, the scores of individual HRS dimensions, as advised by the EFA and CFA results, were computed by dividing the sum of individual item scores by the number of items within each dimension. This approach ensured that dimension score computation aligned with the underlying latent structure of the measure. The total score, representing the entire measure, was determined by dividing the sum of scores of all items within the CFA-confirmed model by the total number of items.

Concurrent validity, a type of criterion validity, assesses whether the extent of the proposed measure associates with another measure that has already been established as valid. The concurrent validity of the HRS was assessed by examining the correlation between individual HRS dimension and total scores and the CD-RISC10 score using Spearman correlation coefficients (rho). It was hypothesised that the scores of those psychological resilience-related dimensions and the total HRS score would have a moderate correlation with the CD-RISC10 score.

Convergent validity assesses the degree of correlation between a test and other tests that measure similar constructs. To examine the convergent validity of the HRS, Spearman correlation coefficients were computed to measure the correlation between HRS dimensions and measures of hope (HHI) and self-efficacy (GES). We hypothesised that there would be a positive and moderate correlation (rho >0.30) between psychological-related HRS dimensions and the HHI and GES.

Discriminant validity, also called divergent validity, was evaluated using Spearman correlation coefficients to assess the correlation between HRS dimensions and measures of maladaptive coping (Brief-COPE), depressive symptoms (PHQ-9), and anxiety (GAD-7). We hypothesised that HRS dimensions would exhibit low (rho<0.3) or statistically insignificant correlations with problem-focused, emotion-focused, and avoidance coping, as well as negative and low correlations with the PHQ-9 and GAD-7, thereby affirming the measure’s specificity in capturing resilience-related constructs. Furthermore, known-groups discriminant validity was assessed by comparing HRS scores between individuals with good and poor physical and mental statuses using Mann–Whitney tests. We hypothesised that individuals with poorer self-perceived physical and mental statuses would have lower HRS dimension scores, particularly those dimensions sharing similar constructs with psychological resilience.

#### Internal consistency reliability

Internal consistency reliability refers to the extent to which the items within a measurement instrument or scale consistently measure the same construct or concept. It assesses the degree of correlation among different items in the same scale or subscale. The internal consistency reliability of each HRS dimension and the entire HRS was assessed in both the EFA and CFA datasets to ensure consistency. A Cronbach’s alpha value of 0.70 or higher was considered acceptable (44).

## Results

### Participant characteristics

Table 1 presents the characteristics of all participants as well as individuals sampled for EFA and CFA, respectively. Approximately 47% of participants were aged 51 years and above, with the majority being females (62.9%) and 72.8% being Chinese. Close to two-thirds of the participants were married (65.7%) and employed (64.9%), while over half of them (52.6%) stayed in 4-room public housing flats built by the Singapore Housing and Development Board. More than four-fifths of individuals (82.5%) perceived their physical health status to be as good or better compared to their peers. This rate increased to 91.1% when considering mental health status. The characteristics of participants sampled for EFA and CFA were found to be comparable, with no significant differences observed in all variables (all *p*-values >0.05).

**Table 1:** The characteristics of participants.

Characteristics	EFA (*n* = 350)	CFA (*n* = 300)	Total (*n* = 650)
Age group			
21–30 years	51 (14.6)	45 (15.0)	96 (14.8)
31–40 years	71 (20.3)	54 (18.0)	125 (19.2)
41–50 years	70 (20.0)	53 (17.7)	123 (18.9)
51–60 years	77 (22.0)	79 (26.3)	156 (24.0)
> = 61 years	81 (23.1)	69 (23.0)	150 (23.1)
Gender			
Male	127 (36.3)	114 (38.0)	241 (37.1)
Female	223 (63.7)	186 (62.0)	409 (62.9)
Chinese	257 (73.4)	216 (72.0)	473 (72.8)
Marital status			
Single	93 (26.6)	79 (26.3)	172 (26.5)
Married	232 (66.3)	195 (65)	427 (65.7)
Widowed/Divorced/Separated	25 (7.1)	26 (8.7)	51 (7.9)
Highest education			
Primary/Lower secondary	33 (9.4)	34 (11.3)	67 (10.3)
Secondary/Post-secondary	157 (44.9)	126 (42)	283 (43.5)
Diploma degree	74 (21.1)	57 (19.0)	131 (20.2)
Bachelor’s degree	86 (24.6)	83 (27.7)	169 (26.0)
Housing type			
Public rental/1–2 room flat	25 (7.1)	17 (5.7)	42 (6.5)
Public 3-room flat	47 (13.4)	36 (12.0)	83 (12.8)
Public 4-room flat	188 (53.7)	154 (51.3)	342 (52.6)
Public 5-room flat & above, including private property	90 (25.7)	93 (31.0)	183 (28.2)
Living alone	28 (8.0)	24 (8.0)	52 (8.0)
Employment status			
Employed/self-employed	230 (65.7)	192 (64.0)	422 (64.9)
Unemployed	20 (5.7)	15 (5.0)	35 (5.4)
Inactive	100 (28.6)	93 (31.0)	193 (29.7)
Perceived money insufficiency	36 (10.3)	34 (11.3)	70 (10.8)
Physical health			
Not as good/Do not know	64 (18.3)	50 (16.7)	114 (17.5)
As good/Better	286 (81.7)	250 (83.3)	536 (82.5)
Mental health			
Not as good/Do not know	37 (10.6)	21 (7.0)	58 (8.9)
As good/Better	313 (89.4)	279 (93)	592 (91.1)

### Item-level descriptive statistics

Table 2 presents the descriptive statistics of individuals items of the 35-item HRS for all participants. Items 11 and 31 were polar opposite items. The distribution of the five response options for each item revealed significant skewness. The “4 = Agree” option was the most frequently selected response (range: 36.6–69.7%) for most of the non-polar opposite items. Conversely, the “1 = Strongly Disagree” option was the least selected response (range: 0–7.9%) for all items except the two polar opposite items, indicating the absence of floor effects. The “5 = Strongly Agree” option ranged from 7.4 to 56.9% with 26 items exceeding 15%, suggesting ceiling effects for these 26 items.

**Table 2:** Item-level descriptive statistics (*N* = 650).

Items	1 = strongly disagree, *n* (%)	2 = disagree, *n* (%)	3 = neither agree nor disagree, *n* (%)	4 = agree, *n* (%)	5 = strongly agree, *n* (%)	Mean ± SD	Media*n* (Q1, Q3)
HRS_1	3 (0.5)	8 (1.2)	44 (6.8)	396 (60.9)	199 (30.6)	4.2 ± 0.7	4 (4, 5)
HRS_2	2 (0.3)	7 (1.1)	43 (6.6)	350 (53.9)	248 (38.2)	4.3 ± 0.7	4 (4, 5)
HRS_3	1 (0.2)	13 (2.0)	112 (17.2)	379 (58.3)	145 (22.3)	4.0 ± 0.7	4 (4, 4)
HRS_4	1 (0.2)	8 (1.2)	47 (7.2)	380 (58.5)	214 (32.9)	4.2 ± 0.6	4 (4, 5)
HRS_5	2 (0.3)	10 (1.5)	46 (7.1)	409 (62.9)	183 (28.2)	4.2 ± 0.6	4 (4, 5)
HRS_6	2 (0.3)	25 (3.9)	120 (18.5)	393 (60.5)	110 (16.9)	3.9 ± 0.7	4 (4, 4)
HRS_7	4 (0.6)	46 (7.1)	72 (11.1)	376 (57.9)	152 (23.4)	4.0 ± 0.8	4 (4, 4)
HRS_8	17 (2.6)	59 (9.1)	103 (15.9)	320 (49.2)	151 (23.2)	3.8 ± 1.0	4 (3, 4)
HRS_9	3 (0.5)	28 (4.3)	109 (16.8)	376 (57.9)	134 (20.6)	3.9 ± 0.8	4 (4, 4)
HRS_10	19 (2.9)	67 (10.3)	134 (20.6)	343 (52.8)	87 (13.4)	3.6 ± 0.9	4 (3, 4)
HRS_11 (reversely worded)	138 (21.2)	159 (24.5)	205 (31.5)	121 (18.6)	27 (4.2)	3.4 ± 1.1	3 (3, 4)
HRS_12	0	18 (2.8)	80 (12.3)	445 (68.5)	107 (16.5)	4.0 ± 0.6	4 (4, 4)
HRS_13	1 (0.2)	14 (2.2)	112 (17.2)	413 (63.5)	110 (16.9)	3.9 ± 0.7	4 (4, 4)
HRS_14	2 (0.3)	29 (4.5)	82 (12.6)	372 (57.2)	165 (25.4)	4.0 ± 0.8	4 (4, 5)
HRS_15	0	12 (1.9)	88 (13.5)	434 (66.8)	116 (17.9)	4.0 ± 0.6	4 (4, 4)
HRS_16	2 (0.3)	27 (4.2)	89 (13.7)	404 (62.2)	128 (19.7)	4.0 ± 0.7	4 (4, 4)
HRS_17	0	12 (1.9)	54 (8.3)	453 (69.7)	131 (20.2)	4.1 ± 0.6	4 (4, 4)
HRS_18	2 (0.3)	21 (3.2)	131 (20.2)	405 (62.3)	91 (14.0)	3.9 ± 0.7	4 (4, 4)
HRS_19	0	8 (1.2)	68 (10.5)	455 (70.0)	119 (18.3)	4.1 ± 0.6	4 (4, 4)
HRS_20	4 (0.6)	68 (10.5)	148 (22.8)	343 (52.8)	87 (13.4)	3.7 ± 0.9	4 (3, 4)
HRS_21	7 (1.1)	36 (5.5)	186 (28.6)	346 (53.2)	75 (11.5)	3.7 ± 0.8	4 (3, 4)
HRS_22	4 (0.6)	29 (4.5)	195 (30.0)	340 (52.3)	82 (12.6)	3.7 ± 0.8	4 (3, 4)
HRS_23	7 (1.1)	47 (7.2)	233 (35.9)	315 (48.5)	48 (7.4)	3.5 ± 0.8	4 (3, 4)
HRS_24	4 (0.6)	19 (2.9)	38 (5.9)	290 (44.6)	299 (46.0)	4.3 ± 0.8	4 (4, 5)
HRS_25	3 (0.5)	12 (1.9)	23 (3.5)	260 (40.0)	352 (54.2)	4.5 ± 0.7	5 (4, 5)
HRS_26	5 (0.8)	17 (2.6)	38 (5.9)	241 (37.1)	349 (53.7)	4.4 ± 0.8	5 (4, 5)
HRS_27	5 (0.8)	9 (1.4)	28 (4.3)	238 (36.6)	370 (56.9)	4.5 ± 0.7	5 (4, 5)
HRS_28	23 (3.5)	48 (7.4)	133 (20.5)	332 (51.1)	114 (17.5)	3.7 ± 1.0	4 (3, 4)
HRS_29	32 (4.9)	47 (7.2)	99 (15.2)	347 (53.4)	125 (19.2)	3.7 ± 1.0	4 (3, 4)
HRS_30	2 (0.3)	13 (2.0)	68 (10.5)	379 (58.3)	188 (28.9)	4.1 ± 0.7	4 (4, 5)
HRS_31 (reversely worded)	51 (7.9)	100 (15.4)	173 (26.6)	245 (37.7)	81 (12.5)	2.7 ± 1.1	2 (2, 3)
HRS_32	3 (0.5)	20 (3.1)	153 (23.5)	362 (55.7)	112 (17.2)	3.9 ± 0.7	4 (3, 4)
HRS_33	2 (0.3)	9 (1.4)	70 (10.8)	413 (63.5)	156 (24.0)	4.1 ± 0.6	4 (4, 4)
HRS_34	4 (0.6)	16 (2.5)	162 (24.9)	355 (54.6)	113 (17.4)	3.9 ± 0.7	4 (3, 4)
HRS_35	1 (0.2)	8 (1.2)	89 (13.7)	418 (64.3)	134 (20.6)	4.0 ± 0.6	4 (4, 4)

### Factorial validity

#### Exploratory factor analysis

The KMO test yielded a value of 0.91 and the Bartlett test of sphericity returned a *p*-value <0.001 for all 35 items based on the EFA dataset (*n* = 350), suggesting these items are not independent and there is substantial correlation present in the data suitable for factor analysis. The initial EFA of the 35 items suggested a four-factor, five-factor, and eight-factor solution based on PA, MAP, and Kaiser’s eigenvalues >1 criterion. However, upon examining the rotated factor loadings, the factors that items loaded onto did not entirely align with the proposed dimensions of health resilience. Specifically, seven items loaded onto more than one factor, and the four items (HRS_8 to HRS_11) proposed for “healthcare affordability” all loaded onto different factors. Furthermore, items proposed for “health literacy” and “perception of medical services” generally loaded onto the same factor, except HRS_7 and HRS_14. Additionally, several items (HRS_28, HRS_29, HRS_31) did not load onto their proposed factors. The content of the respective items loading onto same factors were reviewed.

Items considered theoretically irrelevant to the loaded factors were removed one at a time. Whenever there were any changes to the number of items included for analysis, the item extraction, rotation, and review process was repeated iteratively to streamline the set of items in the HRS. Throughout this process, items that were found to be theoretically irrelevant to the loaded factors (HRS_7 to HRS_9, HRS_11, HRS_14, HRS_19, HRS_28, HRS_29, HRS_31) were removed gradually. Additionally, items loading onto multiple dimensions (e.g., HRS_6 and HRS_20), as well as those with factor loading <0.40 (e.g., HSR_18) or those demonstrating high correlation (rho>0.80) with another item and considered redundant (e.g., HSR_27), were systematically eliminated.

Following this iterative process, a total of 22 items were retained, loading onto five distinct factors, as consistently suggested by both the MAP and Kaiser’s criterion. The five-factor solution accounted for 64.8% of the total variance, with the first factor explaining 34.5% of the total variance (Table 3). The number of items in each factor ranged from three to five. All 22 remaining items exhibited moderate to strong factor loading (>0.50) on their respective factors. The naming of the five factors was refined by the nature and scope of the items. The dimension “Adaptive adjustment” describes a person’s ability to adapt changes or effectively adjust in response to health challenges or changes in circumstances. “Health mindset” refers to an individual’s personality, attitudes, beliefs, perceptions, and strengths that have a protective influence on their responses toward health challenges. “Relational support” refers to the assistance, encouragement, and care provided within interpersonal relationships, such as family, friends, neighbours, or colleagues. “Perceived health access” describes an individual’s view of the accessibility, sufficiency, and quality of health resources that a person can access to manage or overcome health challenges. “Social resourcefulness” refers to an individual’s ability to comprehend, access, and utilise publicly available social care resources to navigate and address health-related challenges.

**Table 3:** Rotated factor loadings for individual items in each factor using the EFA dataset (*n* = 350).

Item		Five-factor solution (22 items)			
	1: Adaptive adjustment	2: Perceived health access	3: Health mindset	4: Relational support	5: Social resourcefulness
HRS_1			0.69		
HRS_2			0.72		
HRS_3			0.56		
HRS_4			0.67		
HRS_5			0.78		
HRS_12		0.64			
HRS_13		0.77			
HRS_15		0.57			
HRS_16		0.88			
HRS_17		0.63			
HRS_10					0.83
HRS_21					0.89
HRS_22					0.80
HRS_23					0.86
HRS_24				0.88	
HRS_25				0.90	
HRS_26				0.92	
HRS_30	0.70				
HRS_32	0.69				
HRS_33	0.62				
HRS_34	0.85				
HRS_35	0.77				
*Unadjusted Eigenvalue*	*7.59*	*2.44*	*1.78*	*1.28*	*1.15*
*% of variance prior rotation*	*34.50*	*11.10*	*8.10*	*5.81*	*5.25*
*% of variance post rotation*	*24.23*	*22.35*	*21.92*	*20.06*	*18.00*

Extraction method: Principal component factoring. Rotation method: Oblique Promax. Loading values less than 0.30 were omitted.

#### Confirmatory factor analysis

**Figure 1:**
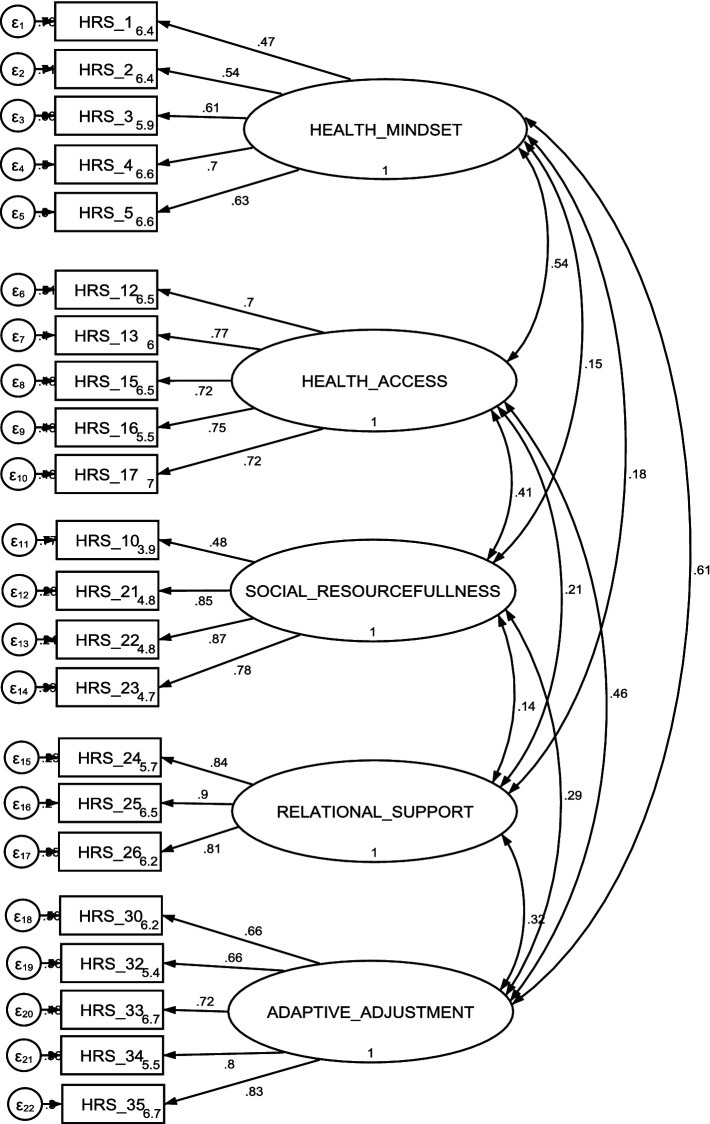
The path diagram for the five-factor CFA model with 22-item HRS: standardised estimates.

The CFA was conducted on the 22 items based on the five-factor structure identified in the final EFA using a separate subset of data (*n* = 300). The standardised factor loading for each item onto their respective latent variable is presented in Figure 1. All items within individual latent variables exhibited factor loadings exceeding 0.45, with 19 out of 22 items displaying factor loadings surpassing 0.55. The χ^2^/df ratio was 1.94 (χ^2^ (199) =385.1, *p* < 0.001) and the values of the goodness fit indexes (RMSEA = 0.05, CFI = 0.94, TLI = 0.93, SRMR = 0.05) suggested a good fit between the hypothesised five-factor model and the observed data.

#### Internal consistency reliability

The internal consistency of the 22-item HRS was acceptable, with Cronbach’s alpha values of 0.89 and 0.85, derived from the EFA and CFA datasets, respectively. Cronbach’s alpha for individual HRS dimensions ranged from 0.76 to 0.90 based on the EFA dataset and from 0.73 to 0.88 based on the CFA dataset. The dimension “Health mindset” exhibited the lowest Cronbach’s alpha values, while “Relational support” demonstrated the highest Cronbach’s alpha values based on both datasets (Table 4).

**Table 4:** Internal consistency reliability (Cronbach’s alpha) of the five HRS dimensions.

Dimension	Item	Cronbach’s alpha (EFA dataset)	Cronbach’s alpha (CFA dataset)
Health mindset	HRS_1. I am determined to maintain /improve my health although it can be difficult	0.76	0.73
	HRS_2. I can keep doing activities that are important to me		
	HRS_3. I am confident I can cope with my health challenges based on my past life experience		
	HRS_4. I believe I can see the bright side of life		
	HRS_5. I believe my health can be improved		
Perceived health access	HRS_12. I can obtain reliable information that I need to promote my health	0.83	0.85
	HRS_13. I can find sufficient information to address my health concerns		
	HRS_15. I can make sense of the information I obtained to address my health concerns		
	HRS_16. I am aware of the health/medical services that could meet my health concerns		
	HRS_17. I can obtain appropriate health /medical services to meet my health needs		
Social resourcefulness	HRS_10. I can obtain financial support (e.g., governmental subsidies or financial assistance schemes, company benefits, family or friends’ financial help) for my medical care if needed	0.87	0.82
	HRS_21. I can obtain appropriate social services (e.g., counselling, caregiver support, family social services) that meet my health concerns if I need		
	HRS_22. I believe I will receive good-quality social care services (e.g., counselling, caregiver support, family social services) to meet my needs		
	HRS_23. I can receive social care services (e.g., counselling, caregiver support, family social services) to meet my health care needs within an acceptable timeframe		
Relational support	HRS_24. I can get support from at least a family member, relative, or friend to address my health concerns and/or needs	0.90	0.88
	HRS_25. I can discuss my health concerns with at least a family member, relative, or friend when needed		
	HRS_26. I have at least a family member, relative, or friend who can take good care of me while I am sick		
Adaptive adjustment	HRS_30. I can encourage myself even if I were to feel challenged by my health	0.82	0.83
	HRS_32. I can adjust my expectations on life when my health worsens		
	HRS_33. I can adopt healthy lifestyles or behaviors to promote my health		
	HRS_34. I can overcome my negative emotions even if I were to feel challenged by my health		
	HRS_35. I can make changes or find ways to accommodate my health condition if needed
22-item HRS		0.89	0.85

#### Concurrent and convergent validity

Table 5 illustrates the Spearman correlation coefficients between five HRS dimensions and CD-RISC10, HHI, and GSE for all participants. Notably, the scores of the three HRS dimensions including “Health mindset,” “Perceived health access,” and “Adaptive adjustment,” as well as the total HRS score exhibited moderate and positive correlation with the “gold standard” resilience measure CD-RISC10 (rho:0.35–0.44), indicating good concurrent validity.

**Table 5:** Correlations between HRS dimensions and validation measures: Spearman’s correlation coefficients (rho).

HRS dimension	Concurrent validity	Convergent validity	
	CD-RISC10	HHI	GSE
Health mindset	0.40*	0.46*	0.39*
Perceived health access	0.35*	0.26*	0.41*
Social resourcefulness	0.08*	−0.01	0.09*
Relational support	0.21*	0.47*	0.05
Adaptive adjustment	0.40*	0.37*	0.30*
HRS total	0.44*	0.42*	0.37*

*p-value < 0.05, HRS: HRS = Health Resilience Scale, CD-RISC10 = Connor-Davidson Resilience Scale 10-item, HHI = Herth Hope Index.

For convergent validity, the scores of the HRS dimensions, including “Health mindset,” “Relational support,” and “Adaptive adjustment,” displayed moderate correlation with HHI score (rho: 0.46, 0.47, and 0.37, respectively). Similarly, the dimensions “Health mindset,” “Perceived health access,” and “Adaptive adjustment” demonstrated moderate correlations with GSE. Surprisingly, “Social resourcefulness” generally exhibited weak or no statistically significant correlation with hope and self-efficacy, and “Relational support” showed no correlation with self-efficacy (Table 5).

#### Divergent validity

The HRS dimensions and total score exhibited weak or nonsignificant yet positive correlations (rho<0.25) with adaptive coping strategies, whereas they displayed weak or nonsignificant negative correlations with maladaptive coping strategies. Moreover, individual HRS dimensions and the total score displayed weak and negative correlations with depressive and anxiety symptoms, as measured by PHQ-9 and GAD-7, respectively (see Table 6). These findings suggested that the HRS dimensions measure distinct constructs in relation to adaptive and maladaptive coping strategies, depression, and anxiety.

**Table 6:** Spearman correlation coefficients (rho) between HRS dimensions and divergent measures.

HRS dimension	Adaptive coping	Maladaptive coping	PHQ-9	GAD-7
Health mindset	0.08*	−0.13*	−0.18*	−0.20*
Perceived health access	0.23*	−0.10*	−0.20*	−0.13*
Social resourcefulness	0.20*	0.03	−0.13*	−0.04
Relational support	−0.02	−0.19*	−0.15*	−0.29*
Adaptive adjustment	−0.03	−0.15*	−0.11*	−0.24*
HRS total	0.15*	−0.13*	−0.21*	−0.25*

*p-value < 0.05, HRS = Health Resilience Scale, PHQ = Patient Health Questionnaire, GAD = Generalised Anxiety Disorder.

Individuals who rated their physical and mental health as “As good” or “Better” generally exhibited higher HRS dimension scores (excluding physical health and “Social resourcefulness”) compared to those who rated their self-perceived physical and mental health as “Not as good” or “Do not know” (Table 7). The distribution of HRS dimension scores between physical and mental health groups was illustrated in Supplementary Figures S1, S2.

**Table 7:** Comparison of HRS dimension scores between known groups.

	Health mindset		Perceived health access		Social resourcefulness		Relational support		Adaptive adjustment	
	Mean ± SD	Median (Q1-Q3)	Mean ± SD	Median (Q1-Q3)	Mean ± SD	Median (Q1-Q3)	Mean ± SD	Median (Q1-Q3)	Mean ± SD	Median (Q1-Q3)
**Physical health**		**<0.001** ^**1**^		**<0.001** ^**1**^		0.928^**1**^		**<0.001** ^**1**^		**<0.001** ^**1**^
Not as good/Do not know (*n* = 114)	3.9 ± 0.5	4.0 (3.6–4.2)	3.8 ± 0.5	4.0 (3.6–4.0)	3.6 ± 0.7	3.8 (3.3–4.0)	4.2 ± 0.8	4.0 (4.0–5.0)	3.8 ± 0.6	4.0 (3.4–4.0)
As good /better (n = 536)	4.2 ± 0.4	4.2 (4.0–4.5)	4.0 ± 0.5	4.0 (3.8–4.2)	3.7 ± 0.7	3.8 (3.3–4.0)	4.4 ± 0.6	4.7 (4.0–5.0)	4.0 ± 0.5	4.0 (3.8–4.4)
**Mental health**		**<0.001** ^**1**^		**0.004** ^**1**^		**0.024** ^**1**^		**<0.001** ^**1**^		**<0.001** ^**1**^
Not as good/Do not know (*n* = 58)	3.8 ± 0.6	4.0 (3.6–4.2)	3.8 ± 0.6	4.0 (3.6–4.0)	3.4 ± 0.8	3.5 (3.0–4.0)	3.7 ± 0.9	4.0 (3.3–4.0)	3.6 ± 0.6	3.8 (3.4–4.0)
As good/better (*n* = 592)	4.2 ± 0.4	4.2 (4.0–4.4)	4.0 ± 0.5	4.0 (3.8–4.2)	3.7 ± 0.7	3.8 (3.3–4.0)	4.5 ± 0.6	4.7 (4.0–5.0)	4.0 ± 0.5	4.0 (3.8–4.4)

^1^ *p*-values were derived using two-sample Wilcoxon rank-sum (Mann–Whitney) tests.

## Discussion

Health resilience has emerged as a crucial concept in promoting well-being and positive health outcomes, particularly in the face of health adversity or chronic conditions. While several studies have explored various aspects of health resilience, there is a lack of a comprehensive, multi-dimensional measure that captures the dynamic and multifaceted nature of this construct. This study aimed to address this gap by developing and validating the HRS, a multi-dimensional measure that is culturally and contextually relevant to the population health strategy of Singapore.

The five-factor structure of the HRS, encompassing “Health mindset,” “Perceived health access,” “Social resourcefulness,” “Relational support,” and “Adaptive adjustment,” aligns with the dynamic and multi-dimensional conceptualisation of health resilience proposed in previous theoretical framework (45–47). However, unlike existing measures that primarily focus on psychological aspects, the HRS captures a broader range of factors, including social support, access to healthcare, and adaptive coping strategies. While some measures have explored specific dimensions of health resilience, such as personality trait (48) or psychological resilience (15, 49), the HRS offers a comprehensive assessment of multiple interrelated factors contributing to an individual’s ability to bounce back from adversity and maintain well-being. This multi-dimensional approach acknowledges the complexity of health resilience and the interplay between various components, which is crucial for developing tailored interventions and support strategies.

Previous resilience instruments have been criticized for their narrow focus or lack of contextual relevance (50). The rigorous development process of the HRS, involving qualitative input from diverse stakeholders and empirical validation, ensures that the scale captures culturally relevant and contextually appropriate aspects of health resilience for community-dwelling adults. The systematic and rigorous development process ensured the robustness and contextual relevance of the HRS, aligning with the dynamic and multifaceted nature of the health resilience paradigm.

The construct validity of the HRS was supported by moderate and positive correlations between “Health mindset,” “Adaptive adjustment,” “Perceived health access,” and the 22-item HRS with psychological resilience, as measured by CD-RISC10, indicating promising concurrent validity. Furthermore, consistent with prior studies (51, 52), the psychological-related dimensions of the HRS exhibited moderate correlations with hope (measured by HHI) and general self-efficacy (measured by GSE), both of which share some similarities in constructs with resilience (53, 54), supporting its convergent validity. However, “Social resourcefulness” and “Relational support” generally exhibited weak or no correlations with psychological-related dimensions of health resilience, which is unsurprising as they do not measure similar constructs as those represented by CD-RISC10, HHI, or GSE (55). Consistent with prior literature, the HRS demonstrated weak correlation with adaptive and maladaptive coping strategies, depressive symptoms, and anxiety (56, 57), thereby supporting its discriminant validity. Furthermore, the HRS scores varied between those with different physical and mental health statuses, confirming its known-group discriminant validity (58).

There are several limitations in the study that need to be addressed. Firstly, participants were primarily recruited from the Northern and Central regions of Singapore using convenience sampling. This recruitment method may introduce selection bias, as individuals who consented to participate in the study might be more health conscious or resilient. Such bias could lead to skewed responses and hinder the generalisability of findings to the entire adult population. Therefore, further validation work is required to corroborate our findings across diverse demographic groups in and potentially across regional states in Southeast Asia. Secondly, the cross-sectional nature of the survey data precludes the assessment of predictive validity, test–retest reliability, and responsiveness of the scale.

To enhance the credibility of the HRS, future research should focus on conducting detailed investigations into its psychometric properties. This includes assessing the scale’s ability to predict health outcomes over time, its stability across different contexts and populations, and its sensitivity to changes. Furthermore, future research efforts should aim to establish a suitable scoring approach and determine threshold scores. This will enable practitioners to effectively assess levels of health resilience and identify individuals requires more support, thus informing targeted interventions and practices. Finally, further studies should explore the applicability and validity of the HRS in other counties, particularly in Southeast Asian regions with cultural and systemic similarities to Singapore, such as Hong Kong, Taiwan, and Malaysia. This would support broader scale adaption and provide deeper insights into health resilience across diverse populations that share similar cultural and social nuances.

## Conclusion

Our findings suggest that the 22-item HRS is a reliable and validated multi-dimensional tool for assessing individual health resilience. It offers valuable insights into the dynamic nature of health resilience, making it an effective scale for guiding initiatives aimed at enhancing health resilience and well-being in community settings. Future research is needed to further establish the psychometric properties of the HRS.

## Data Availability

The raw data supporting the conclusions of this article will be made available by the authors upon written request to the corresponding author.
