# Supplementary material for: Development and validation of the multi-dimensional health resilience scale for community-dwelling adults
Source: Front Public Health. 2025 Feb 12;13:1452738. doi: 10.3389/fpubh.2025.1452738 (PMC11864133; doi:10.3389/fpubh.2025.1452738)
Supplement: Supplementary file 2 [file Data_Sheet_2.pdf]

# Health Resilience Scale for Adults

The following statements are what people may think or feel about their own health or challenges related to their health. **If you are facing or were to face with a significant illness**, please choose the answer that best describes how much you agree or disagree with each statement.

| | Strongly Agree | Agree | Neither agree nor disagree | Disagree | Strongly Disagree |
|---|---|---|---|---|---|
| 1. I am determined to maintain /improve my health although it can be difficult | ☐ | ☐ | ☐ | ☐ | ☐ |
| 2. I can keep doing activities that are important to me | ☐ | ☐ | ☐ | ☐ | ☐ |
| 3. I am confident I can cope with my health challenges based on my past life experience | ☐ | ☐ | ☐ | ☐ | ☐ |
| 4. I believe I can see the bright side of life | ☐ | ☐ | ☐ | ☐ | ☐ |
| 5. I believe my health can be improved | ☐ | ☐ | ☐ | ☐ | ☐ |
| 6. I find ways to address challenges arising from my illness / health concerns | ☐ | ☐ | ☐ | ☐ | ☐ |
| 7. I can seek others' advice or help to achieve better health | ☐ | ☐ | ☐ | ☐ | ☐ |
| 8. I have financial or insurance plan(s) to pay for my health /medical care expenses if needed | ☐ | ☐ | ☐ | ☐ | ☐ |
| 9. I can pay for my daily expenses despite my health /medical care expenses | ☐ | ☐ | ☐ | ☐ | ☐ |
| 10. I can obtain financial support (e.g., governmental subsidies or financial assistance schemes, company benefits, family or friends' financial help) for my medical care if needed | ☐ | ☐ | ☐ | ☐ | ☐ |
| 11. I have to forgo certain treatment (if any) for my health due to affordability considerations | ☐ | ☐ | ☐ | ☐ | ☐ |
| 12. I can obtain reliable information that I need to promote my health | ☐ | ☐ | ☐ | ☐ | ☐ |
| 13. I can find sufficient information to address my health concerns | ☐ | ☐ | ☐ | ☐ | ☐ |
| 14. I can count on my doctor to answer questions about my health | ☐ | ☐ | ☐ | ☐ | ☐ |
| 15. I can make sense of the information I obtained to address my health concerns | ☐ | ☐ | ☐ | ☐ | ☐ |
| 16. I am aware of the health / medical services that could meet my health concerns | ☐ | ☐ | ☐ | ☐ | ☐ |
| 17. I can obtain appropriate health /medical services to meet my health needs | ☐ | ☐ | ☐ | ☐ | ☐ |

| | | | | | |
|---|---|---|---|---|---|
| 18. I can receive health/medical services to meet my health needs within an acceptable timeframe | ☐ | ☐ | ☐ | ☐ | ☐ |
| 19. I believe I will receive good-quality medical services when needed | ☐ | ☐ | ☐ | ☐ | ☐ |
| 20. I am aware of the social care services (e.g., counselling, caregiver support, family social services) that meet my health concerns | ☐ | ☐ | ☐ | ☐ | ☐ |
| 21. I can obtain appropriate social services (e.g., counselling, caregiver support, family social services) that meet my health concerns if I need | ☐ | ☐ | ☐ | ☐ | ☐ |
| 22. I believe I will receive good-quality social care services (e.g., counselling, caregiver support, family social services) to meet my needs | ☐ | ☐ | ☐ | ☐ | ☐ |
| 23. I can receive social care services (e.g., counselling, caregiver support, family social services) to meet my health care needs within an acceptable timeframe | ☐ | ☐ | ☐ | ☐ | ☐ |
| 24. I can get support from at least a family member, relative, or friend to address my health concerns and/or needs | ☐ | ☐ | ☐ | ☐ | ☐ |
| 25. I can discuss my health concerns with at least a family member, relative, or friend when needed | ☐ | ☐ | ☐ | ☐ | ☐ |
| 26. I have at least a family member, relative, or friend who can take good care of me while I am sick | ☐ | ☐ | ☐ | ☐ | ☐ |
| 27. I have at least a family member, relative, or friend who can lend me a listening ear if I need to talk about my health concerns | ☐ | ☐ | ☐ | ☐ | ☐ |
| 28. I tend to reflect on experiences or difficulties related to my health | ☐ | ☐ | ☐ | ☐ | ☐ |
| 29. I focus on finding solutions to better manage my health | ☐ | ☐ | ☐ | ☐ | ☐ |
| 30. I can encourage myself even if I were to feel challenged by my health | ☐ | ☐ | ☐ | ☐ | ☐ |
| 31. I feel it hard to accept an unexpected worsening of my health status | ☐ | ☐ | ☐ | ☐ | ☐ |
| 32. I can adjust my expectations on life when my health worsens | ☐ | ☐ | ☐ | ☐ | ☐ |
| 33. I can adopt healthy lifestyles or behaviors to promote my health | ☐ | ☐ | ☐ | ☐ | ☐ |
| 34. I can overcome my negative emotions even if I were to feel challenged by my health | ☐ | ☐ | ☐ | ☐ | ☐ |
| 35. I can make changes or find ways to accommodate my health condition if needed | ☐ | ☐ | ☐ | ☐ | ☐ |
