# Supplementary figures and images for: Development and validation of the multi-dimensional health resilience scale for community-dwelling adults
Source: Front Public Health. 2025 Feb 12;13:1452738. doi: 10.3389/fpubh.2025.1452738 (PMC11864133; doi:10.3389/fpubh.2025.1452738)

Figure S1. Distribution of of HRS dimension scores between two physical health groups

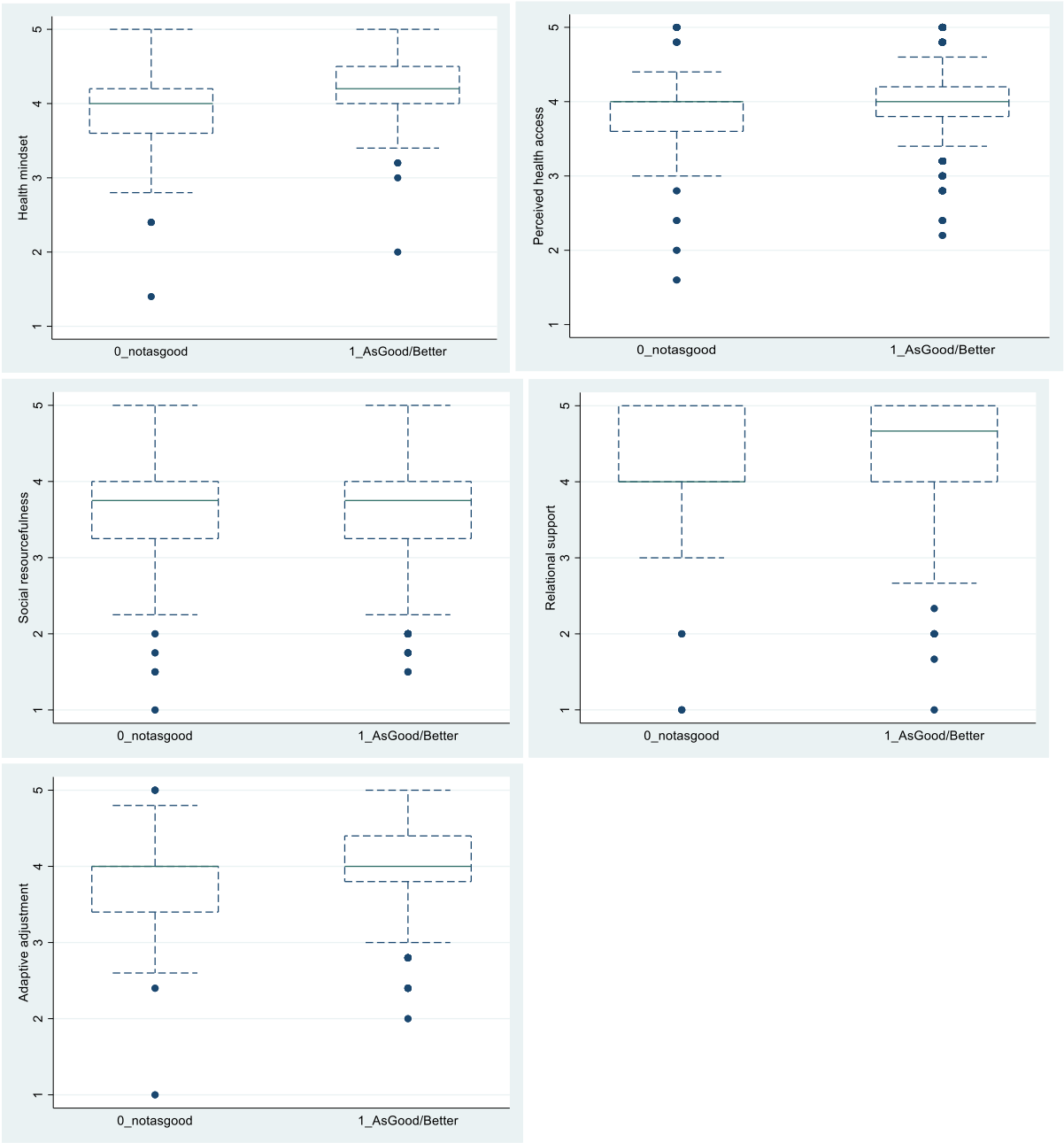

Supplement: Supplementary file 3 [file Image_1.pdf]

Figure S2. Distribution of of HRS dimension scores between two mental health groups

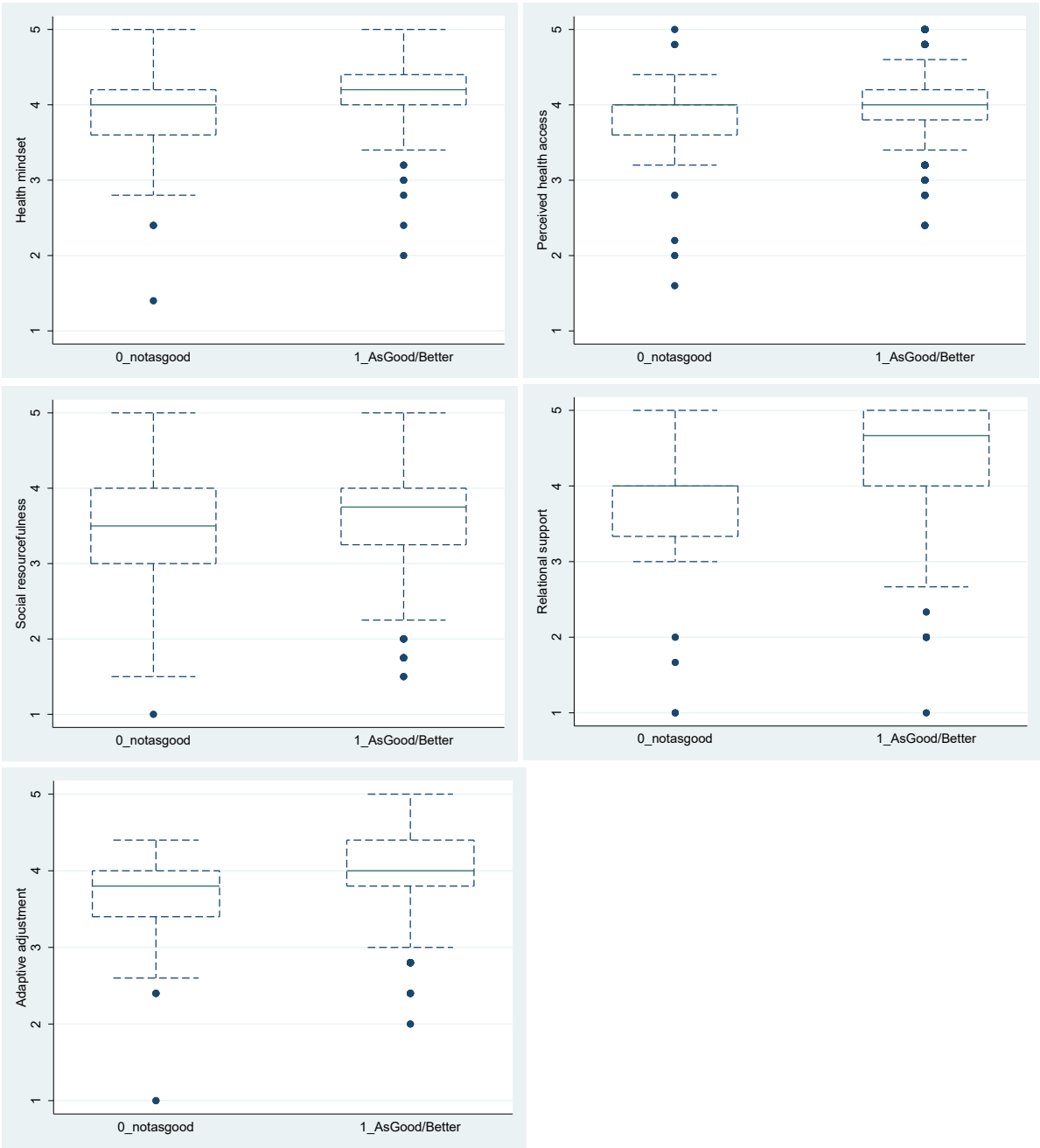

Supplement: Supplementary file 4 [file Image_2.pdf]
